# Association between facial nerve second genu angle and facial canal dehiscence in patients with cholesteatoma: evaluation with temporal multidetector computed tomography and surgical findings^☆^^☆☆^

**DOI:** 10.1016/j.bjorl.2018.03.005

**Published:** 2018-04-14

**Authors:** Asli TanrivermiŞ Sayit, Hediye Pinar Gunbey, Dilek Sağlam, Emre Gunbey, Şemsettin KardaŞ, Çetin Çelenk

**Affiliations:** a Ondokuz Mayis University Faculty of Medicine, Department of Radiology, Samsun, Turkey; b Megapol Hospital, Department of Otorhinolaryngology, İstanbul, Turkey; c Ondokuz Mayis University Faculty of Medicine, Department of Otorhinolaryngology, Samsun, Turkey

**Keywords:** Cholesteatoma, Fascial canal dehiscence, Multidetector computed tomography, Angle at second genu, Colesteatoma, Deiscência do canal facial, Tomografia computadorizada de multidetectores, Ângulo do segundo joelho

## Abstract

**Introduction:**

Otitis media, mastoiditis or the pressure effect of tumorous lesions such as cholesteatoma can be the cause of facial canal dehiscence and facial nerve paralysis. The most common segment involved in dehiscence is the tympanic segment and the second most common is the lateral aspect of the facial canal in the oval window area.

**Objective:**

To determine the prevalence of the facial canal dehiscence and the relationship between the angle at the second genu of the facial nerve and facial canal dehiscence.

**Methods:**

We evaluated the surgical findings in 113 patients who underwent surgery for cholesteatoma. Facial canal dehiscence was detected in 62 of the 113 patients. Patients were divided into two groups: Group 1, with dehiscence of the facial canal and Group 2, without dehiscence of the facial canal.

**Results:**

The mean angles at the second genu of the facial nerve in Groups 1 and 2 were 117.8° ± 9.63° and 114° ± 9.9°, respectively. There was a statistically significant difference between the mean angles at the second genu for the two groups (*p* = 0.04).

**Conclusion:**

In patients with dehiscence of the facial canal, the angle at the second genu was found to be wider than those without dehiscence.

## Resumo

**Introdução:**

Otite média, mastoidite ou a compressão por lesões tumorais como o colesteatoma podem ser a causa da deiscência do canal facial e paralisia do nervo facial. A deiscência ocorre mais frequentemente no segmento timpânico, seguido do aspecto lateral do canal facial na área da janela oval.

**Objetivo:**

Determinar a prevalência da deiscência do canal facial e sua relação com o ângulo no segundo joelho do nervo facial.

**Método:**

Avaliamos os achados cirúrgicos para detecção de deiscência do canal facial em 113 pacientes submetidos à cirurgia de colesteatoma. A deiscência do canal facial foi observada em 62. Os pacientes foram divididos em dois grupos: Grupo 1, com deiscência do canal facial, e Grupo 2, sem deiscência do canal facial.

**Resultados:**

Os ângulos médios no segundo joelho do nervo facial nos grupos 1 e 2 foram 117,8° ± 9,63° e 114° ± 9,9°, respectivamente. Houve diferença estatisticamente significante entre os ângulos médios no segundo joelho para os dois grupos (*p* = 0,04).

**Conclusão:**

Em pacientes com deiscência no canal facial, foi observado que o ângulo do segundo joelho era maior do que naqueles sem deiscência.

## Introduction

Otitis media, mastoiditis or the pressure effect of tumorous lesions such as cholesteatoma can be the cause of facial canal dehiscence and facial nerve paralysis.^1^, ^2^ Facial canal dehiscence can be either congenital or acquired. Congenital facial canal dehiscence is a developmental defect in the bony covering of the facial nerve.^3^ Acquired dehiscence is often associated with atticoantral chronic suppurative otitis media with cholesteatoma.^4^ It may also develop due to long-standing inflammation, prior ear surgery and trauma.^2^

The incidence of facial canal dehiscence has been reported to be between 0.5%^5^ and 74%^6^ based on intraoperative findings. The most common segment involved in dehiscence is the tympanic segment (84.6%), and the second most common is the lateral aspect of the facial canal in the oval window area (69.2%).^7^ The roof of the tympanic segment is very thin, so tumoral lesions such as cholesteatoma or otitis media can easily be the cause of facial canal dehiscence due to the pressure effect. Normally, the angle at the second genu of the facial nerve is 95°–25°. When the angle at the second genu increases, the mastoid part of the facial nerve is displaced posteriorly in the mastoid, away from the chorda tympani and round window.^8^ Because of the increased angle at the second genu, the tympanic segment of the facial nerve has a wider surface area which may lead to dehiscence in a wider surface area in patients with cholesteatoma.^9^, ^10^

A limited number of studies related to the angle at the second genu of the facial nerve are available^8^, ^11^ However, there has been no investigation into the relationship between the angle at the second genu and facial canal dehiscence in patients with cholesteatoma. Here, the aim of the study is to determine the prevalence of facial canal dehiscence and the relationship between the angle at the second genu and facial canal dehiscence in patients with cholesteatoma.

## Methods

### Patients

Institutional Review Board approval was obtained to review the records of all patients who underwent temporal bone Multidetector Computed Tomography (MDCT) between 2011 to 2016 years. The approval protocol number from the Ethics Committee of our institution is B.30.2.ODM.0.20.08/495.

A total of 113 patients (37 female, 76 male) with pathologically proven middle ear cholesteatoma who underwent primary surgery from 2011 to 2016 were enrolled in this study. The medical records of the intraoperative assessment of the facial canal were reviewed using the hospital's database. The facial canal was divided into five segments: geniculate ganglion, tympanic segment, second genu, oval window niche and mastoid (Table 1). Facial canal dehiscence was defined as any discontinuity in the bony structure of the facial canal which resulted in a connection between the facial nerve and any middle ear space or mastoid air cell system.

**Table 1:** Classification of the dehiscent facial canal.

Geniculate ganglion dehiscence	The dehiscence is before the coq.
Tympanic segment dehiscence	The dehiscence is between the second genu and the coq.
Dehiscence at second genu	The dehiscence is located in the second genu very close to the lateral semicircular canal.
Dehiscence of oval window niche	The dehiscence is protruding over the only oval window.
Mastoid segment dehiscence	The dehiscence is after the lower level of the oval window at the mastoid segment.

According to surgical findings, facial canal dehiscence was detected in 62 of the 113 patients. A total of 51 patients with cholesteatoma had no dehiscent facial canal. Patients were divided into two groups: Group 1, with dehiscence of the facial canal and Group 2, without dehiscence of the facial canal. Demographic data of the study population is shown in Table 2.

**Table 2:** Demographic data of the study population.

	Group 1	Group 2
*Number of patients*	62	51
*Gender*		
Female	20 (32.3%)	17 (33.3%)
Male	42 (67.7%)	34 (66.7%)

*Age (years)*		
Mean ± SD	37.3 ± 15.8	34.2 ± 16.7
Min–max	8–75	9–69

SD, standard deviation.

### CT imaging and analysis

High-resolution MDCT imaging was performed with a 16-slice multidetector row computed tomography (CT) scanner (Aquilion 16; Toshiba Medical Systems Corporation, Tokyo, Japan) and a 128-slice multidetector row CT scanner (Discovery, GE Healthcare, Milwaukee, WI, USA). Scanning was performed from the arcuate eminence of the temporal bone to the mastoid tip in the transverse plane with the bone window (2300–2500 HU [Hounsfield Units]) and the soft tissue window (300–500 HU). The scan image plane was parallel to the hard palate. The scanning parameters used were a collimation of 1 mm, Mas = 250, kV = 120, matrix = 512 × 512, algorithm: bony, and reconstruction thickness = 0.5 mm.

**Figure 1:**
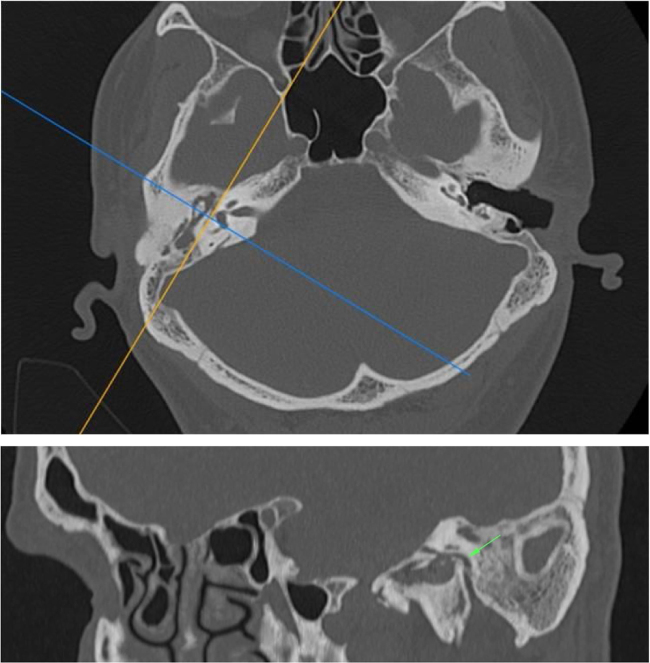
(A) and (B) Reformatted sagittal images were obtained from thin-section axial MDCT images. Angle at the second genu, tympanic, and mastoid segments of the facial nerve (arrow) were demonstrated.

**Figure 2:**
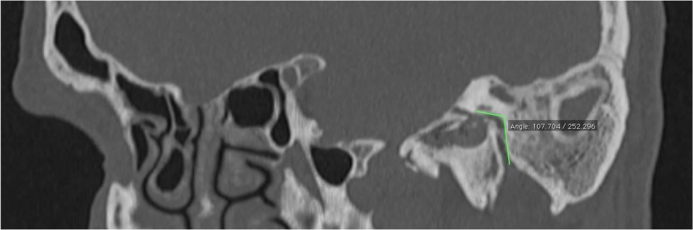
Parallel lines from the tympanic and mastoid segments of the facial nerve were drawn in sagittal reformatted images.

The Digital Imaging and Communications in Medicine (DICOM) files were retrieved from the Picture Archiving and Communication System (PACS) and transferred to the workstation for review; all measurements were done digitally by an experienced radiologist (A.T.S). All patients had preoperative temporal MDCT images taken. Temporal MDCT images with insufficient image quality were not included the study. Reformatted sagittal images were obtained from the thin-section axial MDCT images (Fig. 1A and B). Parallel lines from the tympanic and mastoid segments of the facial nerve were drawn in sagittal reformatted images as shown in the study of Hasaballah et al.^8^ Then, the angle at the second genu of the facial canal of all patients was measured (Fig. 2).

### Statistical analyses

Statistical analyses were done using IBM SPSS Statistics, ver. 21 (IBM Corporation, Armonk, NY, USA). The data is expressed in the mean ± standard deviation (SD) and the median (minimum–maximum). The Shapiro–Wilk test was used to determine the normality in the distribution of the quantitative data. To compare the two independent groups, the Student's *t*-test was used. The interclass correlation coefficient was used to evaluate the reliability of the measurements. A *p*-value of less than 0.05 was considered statistically significant.

## Results

### Patients

**Figure 3:**
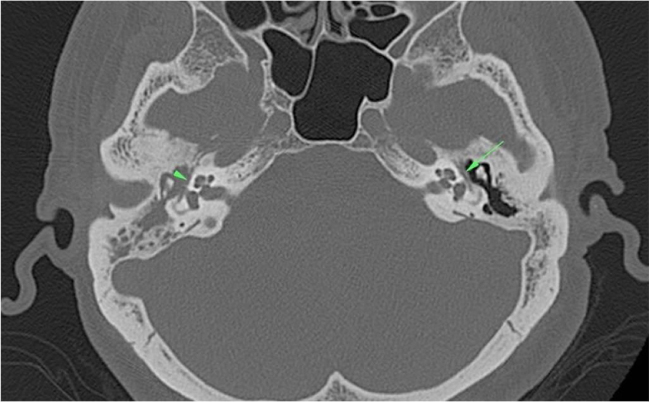
In the right middle ear cavity and mastoid antrum, there was a soft tissue density proven as cholesteatoma. Also, dehiscence in the tympanic segment of the facial canal (arrowhead) was seen on the right side. Tympanic segment of the facial canal (arrow) was intact on the left side.

Dehiscence of the facial canal was observed in 62 (54.8%) of the 113 patients. A total of 52 out of the 62 patients had dehiscence in the tympanic segment of the facial canal (Fig. 3), eight in the mastoid segment, one in the geniculate ganglion level, and one in the genu of the tympanic segment.

### CT imaging and analysis

All the temporal MDCTs enrolled in the study were evaluated by two observers (A.T.S., D.S.) who were blinded to each other's measurement of the angle at the second genu of the facial nerve. The interclass correlation coefficient was 0.997 (95% CI 0.995–0.998, *p* < 0.001), which indicated excellent agreement between the two observers.

The mean angle at the second genu of the facial nerve in Groups 1 and 2 were 117.8° ± 9.63° and 114° ± 9.9°, respectively. There was a statistically significant difference between the mean angles for the two groups (*p* = 0.04) (Table 3).

**Table 3:** Side of the cholesteatoma and the mean angle at second genu of the facial nerve.

	Group 1	Group 2
*Side of the cholesteatoma*		
Right	29 (46.8%)	26 (51%)
Left	33 (53.2%)	25 (49%)

*Angle at second genu*		
Mean ± SD	117.87 ± 9.63	114.04 ± 9.93
Min–max	100–139	93–134

The mean angles at the second genu of the facial nerve in patients with dehiscence of the tympanic and mastoid segment were 117° ± 9° and 121° ± 11°, respectively.

## Discussion

The facial nerve canal begins to develop as a narrow groove or sulcus within the cartilage of the otic capsule. Ossification then starts from the apical otic ossification center at 21 gestational weeks and from the canalicular ossification center at 26 gestational weeks near the stapedius muscle. The two centers fuse near the region of the oval window until one year after birth.^2^, ^12^ From an anatomical and radiological standpoint, the facial canal is completely developed by four years of age.^13^ However, middle ear inflammations can affect the development of the facial canal in children.^12^ Also, facial canal dehiscence may develop due to prior ear surgery, trauma and the pressure effect from tumorous lesions.^2^

The incidence of facial canal dehiscence was reported in a relatively wide range from 0.5%^5^ to 74%^6^ based on histologic and surgical studies. Dehiscence of the facial canal must be at least 1 mm in size to be detected during surgery.^12^ However, the incidence of facial canal dehiscence is higher in histological studies, since it can be detected in microdehiscences of less than 1 mm in cadaveric studies.^2^ Takashi and Sando found that 40% of all dehiscences were detected on the inferior to inferomedial aspect of the facial canal in the posterior half of the oval window area.^6^ Baxter revealed that 85% of all dehiscences occurred through the inferior surface of the tympanic segment toward the oval window niche.^14^ In fact, it is not possible to see these dehiscent areas with routine otologic surgery.

Selesnick and Lynn-Macrae evaluated the incidence of facial canal dehiscence in a study of 67 cholesteatoma cases.^7^ They found facial canal dehiscences in 30% of the initial surgical procedures and in 35% of the revision procedures. They reported that the tympanic segment was the most common region (81%) of facial canal dehiscence occurrence.^7^ Kim et al. reviewed 152 patients for intraoperative findings of facial canal dehiscence and observed facial canal dehiscence in 13 (8.6%) of 152 patients.^2^ The most commonly involved region was the tympanic segment, which accounted for 84.6% of the incidence of canal dehiscence. Facial canal dehiscence was noted on the lateral aspect of the facial canal in the oval window area at an incidence of 69.2%.^2^ In our study, 113 patients who had pathologically proven middle ear cholesteatoma were included, and 62 of 113 patients had surgically confirmed facial canal dehiscence while 51 did not. The incidence of facial nerve canal dehiscence in patients with cholesteatoma was 54.8%. Dehiscence was found in 52 of 62 cases in the tympanic segment of the facial canal, eight were found in the mastoid segment, one was found in the geniculate ganglion, and the other was found in the second genu.

The first part of the facial canal is the labyrinthine segment of the nerve. It extends from the fundus of the internal auditory canal to the geniculate ganglion. At this level, the direction of the nerve reverses itself, executing a hairpin turn so that it runs posteriorly. This is the ‘first turn’ of the facial nerve. The second part of the facial nerve is the tympanic segment which runs posterior-superior to the cochleariform process, superior and lateral to the oval window, and then continues inferior to the lateral semicircular canal. At the pyramidal process, the tympanic segment turns inferiorly at a 95°–125° angle (at the second genu) to become the mastoid or vertical segment.^9^, ^10^ Hasaballah et al. evaluated the facial nerve course in a study of patients with cochlear implants.^8^ In these patients, the mean angle at the second genu was found to be 105.8° ± 13.2°. Yadav et al. examined the anatomy of the tympanomastoid segment of the facial nerve in 25 adult human wet cadaveric temporal bones under a microscope.^15^ They reported the angle at the second genu between the tympanic and mastoid segment to be 110° in 64%, 95° in 32% and 125° in 4% of the specimens. In our study, the angle at the second genu was measured in the oblique sagittal sections of the temporal MDCT as shown by Hasaballah and colleagues. The mean angle at the second genu in patients with and without facial canal dehiscence was 117.8° ± 9.63° and 114° ± 9.9°, respectively. Also, there was a statistically significant difference between the mean angles of the two groups. When the two groups were statistically analyzed, the angle at the second genu was found to be significantly higher in patients with dehiscent facial canals.

When the angle at the second genu increases, the mastoid segment of the facial nerve is displaced posteriorly in the mastoid, away from the chorda tympani and round window. The round window becomes more visible, which means that the tympanic segment of the facial nerve has a wider surface area.^8^ The tympanic segment roof is normally very thin, causing a tendency for dehiscence in the tympanic segment of the facial nerve when there is an increased angle at the second genu (≥117.87 ± 9.63), especially in middle ear pathologies such as cholesteatoma or otitis.

Facial nerve damage in ear surgeries is a serious complication for the otologic surgeon. The incidence of iatrogenic facial nerve injury in all otologic surgical procedures is 0.6%–3.6%, and is even higher in revision surgeries (4°–10%).^2^ The localization of cholesteatoma, relation with ossicular chain, erosion in adjacent bone structures, presence of complications and fascial canal dehiscence can be evaluated with preoperative temporal MDCT. However, it is not easy to see facial canal dehiscence with temporal MDCT when considering microdehiscences. Rogha et al. found there to be a very low radiosurgical correlation (kappa statistic, *k* = 0.2) in detecting facial canal dehiscence.^16^ Therefore, the angle at the second genu of the facial nerve can be measured using temporal MDCT in cases where facial canal dehiscence is suspected. It should be considered that facial canal dehiscence may be present in patients with cholesteatoma with an increased angle at the second genu of the facial canal.

## Conclusion

The prevalence of facial canal dehiscence was 54.8% in patients with cholesteatoma and was most commonly seen in the tympanic segment of the facial canal. Additionally, the angle at the second genu of the facial nerve in patients with dehiscent facial canal was greater than in those without dehiscence. An increased angle at the second genu can be the cause of the wider surface area of the tympanic segment. Thus, patients with an increased angle at the second genu may be more prone to dehiscence of the facial canal.

## Conflicts of interest

The authors declare no conflicts of interest.

## References

[1] Perez B., Campos M.E., Rivero J., Lopez Campos D., López-Aguado D. (1997). Incidence of dehiscences in the fallopian canal. Int J Pediatr Otorhinolaryngol.

[2] Kim C.W., Rho Y.S., Ahn H.Y., Oh S.J. (2008). Facial canal dehiscence in the initial operation for chronic otitis media without cholesteatoma. Auris Nasus Larynx.

[3] Proctor B., Nager G.T. (1982). The facial canal: normal anatomy, variations and anomalies. I. Normal anatomy of the facial canal. Ann Otol Rhinol Laryngol Suppl.

[4] Lin J.C., Ho K.Y., Kuo W.R., Wang L.F., Chai C.Y., Tsai S.M. (2004). Incidence of dehiscence of the facial nerve at surgery for middle ear cholesteatoma. Otolaryngol Head Neck Surg.

[5] Derlacki E.L., Shambaugh G.E., Harrison W.H. (1957). The evolution of a stapes mobilization technique. Laryngoscope.

[6] Takahashi H., Sando I. (1992). Facial canal dehiscence: histologic study and computer reconstruction. Ann Otol Rhinol Laryngol.

[7] Selesnick S.H., Lynn-Macrae A.G. (2001). The incidence of facial nerve dehiscence at surgery for cholesteatoma. Otol Neurotol.

[8] Hasaballah M.S., Hamdy T.A. (2014). Evaluation of facial nerve course, posterior tympanotomy width and visibility of round window in patients with cochlear implantation by performing oblique sagittal cut computed tomographic scan temporal bone. Egypt J Otolaryngol.

[9] Măru N., Cheiţă A.C., Mogoantă C.A., Prejoianu B. (2010). Intratemporal course of the facial nerve: morphological, topographic and morphometric features. Rom J Morphol Embryol.

[10] Gupta S., Mends F., Hagiwara M., Fatterpekar G., Roehm P.C. (2013). Imaging the facial nerve: a contemporary review. Radiol Res Pract.

[11] Li J.M., Xu W.B., Zhong J.W., Wu H.Y., Dai W.C. (2016). CT study on the development of facial nerve canal in children. Zhonghua Er Bi Yan Hou Tou Jing Wai Ke Za Zhi.

[12] Nomiya S., Kariya S., Nomiya R., Morita N., Nishizaki K., Paparella M.M. (2014). Facial nerve canal dehiscence in chronic otitis media without cholesteatoma. Eur Arch Otorhinolaryngol.

[13] Weiglein A.H. (1996). Postnatal development of the facial canal. An investigation based on cadaver dissections and computed tomography. Surg Radiol Anat.

[14] Baxter A. (1971). Dehiscence of the fallopian canal: an anatomical study. J Laryngol Otol.

[15] Yadav S.P., Ranga A., Sirohiwal B.L., Chanda R. (2006). Surgical anatomy of tympano-mastoid segment of facial nerve. Indian J Otolaryngol Head Neck Surg.

[16] Rogha M., Hashemi S.M., Mokhtarinejad F., Eshaghian A., Dadgostar A. (2014). Comparison of preoperative temporal bone CT with intraoperative findings in patients with cholesteatoma. Iran J Otorhinolaryngol.

